# The effect of moving to East Village, the former London 2012 Olympic and Paralympic Games Athletes' Village, on physical activity and adiposity (ENABLE London): a cohort study

**DOI:** 10.1016/S2468-2667(19)30133-1

**Published:** 2019-07-22

**Authors:** Claire M Nightingale, Elizabeth S Limb, Bina Ram, Aparna Shankar, Christelle Clary, Daniel Lewis, Steven Cummins, Duncan Procter, Ashley R Cooper, Angie S Page, Anne Ellaway, Billie Giles-Corti, Peter H Whincup, Alicja R Rudnicka, Derek G Cook, Christopher G Owen

**Affiliations:** a Population Health Research Institute, St George's, University of London, London, UK; b Department of Public Health, Environments and Society, London School of Hygiene & Tropical Medicine, London, UK; c Centre for Exercise, Nutrition and Health Sciences and National Institute for Health Research Bristol Biomedical Research Centre (Nutrition Theme), University Hospitals Bristol NHS Foundation Trust, University of Bristol, Bristol, UK; d Medical Research Council/Scottish Government Chief Scientist Office Social and Public Health Sciences Unit, University of Glasgow, Glasgow, UK; e National Health and Medical Research Council Centre of Research Excellence in Healthy Liveable Communities, RMIT University, Melbourne, VIC, Australia

## Abstract

**Background:**

The built environment can affect health behaviours, but longitudinal evidence is limited. We aimed to examine the effect of moving into East Village, the former London 2012 Olympic and Paralympic Games Athletes' Village that was repurposed on active design principles, on adult physical activity and adiposity.

**Methods:**

In this cohort study, we recruited adults seeking new accommodation in East Village and compared physical activity and built environment measures with these data in control participants who had not moved to East Village. At baseline and after 2 years, we objectively measured physical activity with accelerometry and adiposity with body-mass index and bioimpedance, and we assessed objective measures of and participants' perceptions of change in their built environment. We examined the change in physical activity and adiposity between the East Village and control groups, after adjusting for sex, age group, ethnicity, housing tenure, and household (as a random effect).

**Findings:**

We recruited participants for baseline assessment between Jan 24, 2013, and Jan 7, 2016, and we followed up the cohort after 2 years, between Feb 24, 2015, and Oct 24, 2017. At baseline, 1819 households (one adult per household) consented to initial contact by the study team. 1278 adults (16 years and older) from 1006 (55%) households participated at baseline; of these participants, 877 (69%) adults from 710 (71%) households were assessed after 2 years, of whom 441 (50%) participants from 343 (48%) households had moved to East Village. We found no effect associated with moving to East Village on daily steps, the time spent doing moderate-to-vigorous physical activity (either in total or in 10-min bouts or more), daily sedentary time, body-mass index, or fat mass percentage between participants who had moved to East Village and those in the control group, despite sizeable improvements in walkability and neighbourhood perceptions of crime and quality among the East Village group relative to their original neighbourhood at baseline.

**Interpretation:**

Despite large improvements in neighbourhood perceptions and walkability, we found no clear evidence that moving to East Village was associated with increased physical activity. Improving the built environment on its own might be insufficient to increase physical activity.

**Funding:**

National Institute for Health Research and National Prevention Research Initiative.

## Introduction

Physical inactivity is a leading cause of premature mortality that is responsible for more than 5 million deaths worldwide per year.^1^ Low amounts of physical activity are associated with numerous chronic diseases in adulthood.^2^ In the UK, a third of men and 40% of women do not meet physical activity recommendations,^3^ which has led to initiatives to increase population-level physical activity that have become enshrined within public health policy.^4^ Dose-response meta-analyses^2^ suggest that even small increases in physical activity would confer substantial health benefits, particularly in cardiovascular and diabetes-related outcomes.

The built environment might affect the amount of physical activity that people do.^5^ Improved walkability (ie, the ability to walk in an area), and improved access to green space, public transport, and recreational facilities might plausibly increase the amount of physical activity.^6^ However, evidence on the effectiveness of these measures is reliant on cross-sectional studies,^7^ which are prone to selection bias, in which those who live in less walkable neighbourhoods are intrinsically different to those who do not. Such studies make it difficult to establish temporality or to infer causal effects. Longitudinal studies have sought to establish causal associations, but the number of studies is small. Systematic reviews^8^ of studies to date have found the evidence less convincing. Specific weaknesses in methods have been that most studies compare movers with non-movers, who could be inherently different,^8^ and that studies^9^ often rely on self-reported physical activity, which is prone to measurement error and reporting bias. These issues with research reliability could affect health outcomes at a population level.^10^ Despite these issues, community-wide built-environment interventions remain attractive, given their potential population reach.

**Research in context**

**Evidence before this study**

A comprehensive systematic search of the literature was completed in 2017 and published in 2018, as cited in our study. This search was registered with the International Prospective Register of Systematic Reviews (number CRD42017077681), and it included a search of the electronic databases MEDLINE, The Cochrane Library, Embase, CINAHL, SPORTDiscus, PsycINFO, InformIT, Avery, and the Royal Institute of British Architects for peer-reviewed papers, and The Grey Literature Report, ProQuest Dissertations, and Theses Global for grey literature. This search also identified articles by forward citation tracking of included publications. The search revealed a growing body of evidence that the local built environment affects the physical activity of individuals living in the neighbourhood. However, much of the evidence was based on cross-sectional studies, and longitudinal evidence has been less supportive of this hypothesis. Positive findings can be attributed to bias, where those who choose to move to potentially better areas are inextricably different from those who do not, an issue that few studies have assessed.

**Added value of this study**

The repurposed East Village, formerly the London 2012 Olympic and Paralympic Athletes' Village, offered a unique opportunity to run a natural experiment. We assessed the effect of moving to a newly built environment that was designed for active living on physical activity and other health-related outcomes. We found improvements in objective measures of the built environment and substantive gains in neighbourhood perceptions associated with moving to East Village. However, we found no appreciable improvements in accelerometry-assessed physical activity or adiposity at a 2-year follow-up.

**Implications of all the available evidence**

The null findings from our study provide an important corrective to earlier studies, and they suggest that changes to the built environment alone might not be sufficient to increase population-level physical activity. However, we acknowledge that a cohort of individuals seeking to move to East Village, an active permissible space built on active design principles, were targeted for the study, and their activity patterns might differ from the population at large. Hence, this possible bias might imply restrictions to the generalisability of our findings.

High-quality evidence is needed to evaluate the effect of the built environment on health behaviours, particularly physical activity. Research should make use of natural experiments in which the population effects following a notable change in built environment can be examined.^11^ The rapid creation and occupancy of East Village—formerly the London 2012 Olympic and Paralympic Athletes' Village, a purpose-built mixed-use residential development that was specifically designed to encourage healthy active living by improving walkability and access to public transport—provided the opportunity for a natural experiment.^12^ We aimed to examine changes in objectively measured physical activity and adiposity and the objective and subjective quality of the built environment among adults who moved to different tenured accommodation in East Village compared with those who did not.

## Methods

### Study design and participants

In this cohort study, the Examining Neighbourhood Activities in Built Living Environments in London (ENABLE London) study, we recruited individuals and families from those seeking new accommodation in East Village. East Village includes several features that could plausibly have a beneficial effect on the physical activity patterns of occupants.^12^ These features include more equitable access to improved public transport (being closer to public transport links) and active travel opportunities (increased walkability, access to green space, and cycle paths).^13^, ^14^ East Village is also situated close to leisure-time physical activity-permissive features, including parkland, street furniture, pedestrianised areas, and major sporting facilities. It is also situated within walking distance of retail outlets.^13^, ^14^ Hence, collectively, East Village has several activity-permissive features that could plausibly increase physical activity by encouraging time spent outdoors.^15^ There were three types of housing tenure being sought by participants: social (provided by the local authority or housing association at subsidised rates), intermediate (a mixture of shared ownership, shared equity, and partly subsidised affordable rental properties) and market rent (ie, with no subsidy).

Participants living in social housing were largely from the London borough of Newham, whereas participants seeking intermediate and market rent accommodation had a greater London geographical diversity, although most of these participants were from east London. We recruited participants from the three housing groups at baseline in three phases, determined by the order of availability (ie, social, intermediate, then market rent housing). Full details of the study recruitment process were published previously.^12^ Participants were followed up 2 years after baseline analyses. Full ethical approval was obtained from the City Road and Hampstead Ethical Review Board (12/LO/1031). All participants provided written informed consent.

### Procedures

Participants wore a hip-mounted ActiGraph GT3X+ accelerometer (ActiGraph; Pensacola, FL, USA) during waking hours for 7 days. Accelerometers provided daily measures of steps (the primary outcome), time spent in moderate-to-vigorous physical activity (MVPA), and time spent sedentary, based on established thresholds (sedentary, <100 counts per min; moderate physical activity, ≥1952 counts per min).^16^ Periods of time in which the accelerometers were not worn were defined as 60 min or more of zero values, allowing for a 2-min spike tolerance, to provide the daily wear time. We excluded days of accelerometer data in which the duration of registered wear time accumulated was less than 540 min. Participants with at least 1 day of data at both baseline and follow-up were included in analyses.

Height was measured to the last complete mm with a portable stadiometer. A Tanita SC-240 Body Composition Analyzer (Tanita; Tokyo, Japan) was used to measure weight to the nearest kg and leg-to-leg bioelectrical impedance, from which fat-free mass and fat mass were estimated. Body-mass index (BMI) was calculated as weight (kg)/height (m^2^), and fat mass percentage was calculated as 100 × (fat mass in kg/weight in kg).

Laptop-based self-completion questionnaires were used to collect data on age, sex, self-defined ethnicity, work status, occupation and number of children in the household. Participant ethnicities were categorised as white, Asian, black, mixed, or other, and mixed and other ethnicities were combined in the analyses. Occupation-based socioeconomic status was coded with the National Statistics Social-Economic Coding to categorise participants into higher managerial or professional occupations, intermediate occupations, or routine or manual occupations. An additional economically inactive category included those who were seeking employment, unable to work because of disability or illness, retired, or looking after home and family, and students. Two neighbourhood perception scores measuring participants' perceptions of crime (ie, vandalism, feeling unsafe to walk in neighbourhood, presence of threatening groups) and neighbourhood quality (ie, accessible features, attractiveness, and enjoyment of living in neighbourhood), were derived at baseline with exploratory factor analysis on 14 neighbourhood perception items in the questionnaire,^17^ and the same items were used to obtain scores at follow-up (appendix pp 1–2).

Residential built-environment characteristics were derived with Geographic Information Systems data at baseline and at follow-up for those households in the Greater London area. These characteristics included street-path network distance (as opposed to straight line distance) from home to the closest park (with data from the London Development Database),^18^ public transport access,^19^ and measures of neighbourhood walkability, which is a relative index derived from a composite score of land use mix (the distribution of residential, commercial, office, entertainment, and institutional building footprints in the area), street connectivity (from the number of junctions with three or more branch roads), and residential density within a km^2^ street network of the home address (appendix pp 1–2). Questionnaires and physical activity and adiposity protocols were identical at baseline and follow-up, and these were administered during home visits by a team of trained fieldworkers.

### Statistical analysis

We quantified changes between baseline and follow-up in objective (Geographic Information Systems-based) and perceived (self-reported) environment metrics. We fitted multilevel linear regression models to examine the effect of moving to East Village on the amount of physical activity and on adiposity compared with controls who did not live in East Village. The primary outcome was specified a priori to be change in daily steps in those who moved to East Village compared with control group participants at 2-year follow-up; secondary outcomes were the time spent in MVPA (total and in bouts of at least 10 min per week), daily sedentary time, BMI, fat mass percentage, and neighbourhood characteristic score.^12^ The study was powered to detect a difference in the primary outcome of 750 steps.^12^

We regressed average daily steps (adjusted for day of week, day order, and month) at follow-up on average daily steps at baseline, adjusting for the East Village or control group as a fixed effect and household as a random effect, to allow for household clustering. Because of baseline differences in sociodemographic factors, we examined further adjustment for participant characteristics, including sex, age group, and ethnic group (white, Asian, black, and mixed or other) in a prespecified analysis. We used stratified models by housing tenure to examine the effect of moving to East Village in the different housing groups; work status, occupation, and number of children were not adjusted for because these were strongly related to housing status. We also included an interaction term between intervention group and housing tenure group.

We used prespecified sensitivity analyses for daily steps, the primary outcome, by restricting the sample to those with at least 4 days of at least 540 min recording at baseline and follow-up; repeating analyses for weekdays only and weekend days only, since these restrictions will modify exposure to the residential built environment; comparing East Village participants with controls who remained at their baseline address at follow-up and controls who had moved elsewhere; and multiple imputation methods to asses the effects of missing accelerometry data at follow-up. All analyses were done with Stata/SE version 15.

### Role of the funding source

The funders of the study had no role in study design, data collection, data analysis, data interpretation, or writing of the report. The corresponding author had full access to all the data in the study and had final responsibility for the decision to submit for publication.

## Results

We recruited participants for baseline assessment between Jan 24, 2013, and Jan 7, 2016, in three phases, determined by the order of housing availability. We followed up the cohort after approximately 2 years, between Feb 24, 2015, and Oct 24, 2017.

At baseline, 1819 households (one adult per household) consented to initial contact by the study team. 1278 adults (16 years and older) from 1006 (55%) households participated at baseline. Of the participating households, 392 (39%) households were seeking social housing, 421 (42%) households were seeking intermediate housing, and 193 (19%) households were seeking market rent housing. The proportion of participants who completed questionnaires was similar in those seeking market rent (58%) and intermediate housing (57%), but it was slightly lower in the social housing group (52%). At the 2-year follow-up, 877 (69%) adults from 710 (71%) households were assessed again (after a median of 107 weeks; range 82–148); 441 (50%) participants from 343 (48%) households had moved and were living in East Village at follow-up. Baseline and follow-up adiposity data were available for 822 (94%) participants, and baseline and follow-up physical activity data were available for 762 (87%) participants. At follow-up, 353 (46%) participants had recorded 7 days of physical activity data (≥6 days, 70%; ≥5 days, 84%; ≥4 days, 94%; ≥3 days, 96%; ≥2 days, 95%). The proportions of participants with several days of physical activity data at baseline were similar (7 days, 45%; ≥6 days, 68%; ≥5 days, 81%; ≥4 days, 91%; ≥3 days, 94%; ≥2 days, 97%). We found no differences in age, sex, or ethnic group between those followed up and those not followed up (appendix pp 3–4), although those who were followed up had a slightly higher socioeconomic status and recorded more sedentary time at baseline (585 min) compared with those not followed up (562 min).

Baseline characteristics for those with data at follow-up are shown in table 1. In the social housing group, age, sex, socioeconomic status, and number of children in the household were similar between those living in East Village and control participants, although the East Village group had a higher proportion of participants of black African or Caribbean ethnic origin and a lower proportion of participants of Asian ethnic origin than the control group. In the intermediate group, the East Village group were younger, less likely to be female, more likely to be white and economically active with no children in the household compared with control group participants. In the market rent group, age, sex, ethnicity, socioeconomic status, and number of children in the household were similar between East Village and control groups. For all housing groups combined, the proportion of women in the two groups were similar, but participants in the East Village group were younger, more likely to be of black African or Caribbean ethnicity, less likely to be of Asian ethnicity, and less likely to be in higher managerial or professional occupations compared with control participants. Overall, control participants showed more baseline daily steps (9192 steps) than East Village group (8644 steps), although we found no differences when stratifying by housing group. Although we found no overall difference in baseline adiposity, control participants in the intermediate group had higher BMIs and fat mass percentages.

**Table 1:** Baseline demographic characteristics, physical activity, and adiposity outcomes, by housing group, for participants who completed 2-year follow-up

		**All housing groups (n=877)**			**Social housing group (n=344)**			**Intermediate housing group (n=377)**			**Market rent housing group (n=156)**		
		Control (n=436)	East Village (n=441)	p value	Control (n=124)	East Village (n=220)	p value	Control (n=203)	East Village (n=174)	p value	Control (n=109)	East Village (n=47)	p value
Age, years		··	··	0·01	··	··	0·27	··	··	<0·0001	··	··	0·07
	16–24	75 (17%)	104 (24%)	··	18 (15%)	47 (21%)	··	30 (15%)	38 (22%)	··	27 (25%)	19 (40%)	··
	25–34	185 (42%)	194 (44%)	··	32 (26%)	61 (28%)	··	100 (49%)	113 (65%)	··	53 (49%)	20 (43%)	··
	35–49	138 (32%)	123 (28%)	··	66 (53%)	95 (43%)	··	61 (30%)	22 (13%)	··	11 (10%)	6 (13%)	··
	≥50	38 (9%)	20 (5%)	··	8 (6%)	17 (8%)	··	12 (6%)	1 (1%)	··	18 (17%)	2 (4%)	··
Sex		··	··	0·79	··	··	0·75	··	··	0·02	··	··	0·53
	Female	248 (57%)	247 (56%)	··	91 (73%)	158 (72%)	··	107 (53%)	70 (40%)	··	50 (46%)	19 (40%)	··
	Male	188 (43%)	194 (44%)	··	33 (27%)	62 (28%)	··	96 (47%)	104 (60%)	··	59 (54%)	28 (60%)	··
Ethnic group		··	··	0·0001	··	··	<0·0001	··	··	0·0002	··	··	0·42
	White	225 (52%)	212 (48%)	··	25 (20%)	38 (17%)	··	122 (60%)	139 (80%)	··	78 (72%)	35 (74%)	··
	Black	81 (19%)	131 (30%)	··	40 (32%)	120 (55%)	··	32 (16%)	9 (5%)	··	9 (8%)	2 (4%)	··
	Asian	91 (21%)	56 (13%)	··	47 (38%)	31 (14%)	··	35 (17%)	18 (10%)	··	9 (8%)	7 (15%)	··
	Other	39 (9%)	42 (10%)	··	12 (10%)	31 (14%)	··	14 (7%)	8 (5%)	··	13 (12%)	3 (6%)	··
Occupation, based on National Statistics Social-Economic Coding		··	··	<0·0001	··	··	0·12	··	··	0·007	··	··	0·53
	Higher managerial or professional	246 (57%)	179 (41%)	··	24 (20%)	23 (11%)	··	146 (73%)	124 (72%)	··	76 (70%)	32 (68%)	··
	Intermediate	54 (12%)	69 (16%)	··	16 (13%)	27 (12%)	··	22 (11%)	34 (20%)	··	16 (15%)	8 (17%)	··
	Routine or manual	44 (10%)	56 (13%)	··	26 (21%)	46 (21%)	··	13 (6%)	10 (6%)	··	5 (5%)	0	··
	Economically inactive	89 (21%)	133 (30%)	··	57 (46%)	121 (56%)	··	20 (10%)	5 (3%)	··	12 (11%)	7 (15%)	··
Number of children in household		··	··	0·10	··	··	0·21	··	··	0·004	··	··	0·65
	None	264 (61%)	238 (54%)	··	15 (12%)	42 (19%)	··	151 (74%)	153 (88%)	··	98 (90%)	43 (91%)	··
	One	80 (18%)	85 (19%)	··	38 (31%)	68 (31%)	··	34 (17%)	15 (9%)	··	8 (7%)	2 (4%)	··
	Two or more	92 (21%)	118 (27%)	··	71 (57%)	110 (50%)	··	18 (9%)	6 (3%)	··	3 (3%)	2 (4%)	··
Physical activity		n=405	n=403	··	n=112	n=199	··	n=189	n=164	··	n=104	n=40	··
	Daily steps	9192 (3284)	8644 (3104)	0·01	7707 (3069)	7730 (3345)	0·95	9639 (3224)	9592 (2584)	0·88	9980 (3128)	9304 (2468)	0·22
	Daily moderate-to-vigorous physical activity, min	61 (26)	58 (25)	0·06	50 (25)	50 (25)	0·97	63 (24)	66 (22)	0·31	70 (26)	65 (21)	0·32
	Daily moderate-to-vigorous physical activity in ≥10-min bouts, min	22 (20)	19 (18)	0·02	12 (14)	12 (13)	0·85	23 (19)	25 (19)	0·45	30 (21)	28 (21)	0·70
	Daily sedentary time, min	588 (81)	583 (84)	0·36	544 (84)	545 (83)	0·91	598 (75)	617 (68)	0·01	616 (67)	627 (74)	0·42
Adiposity		n=428	n=435	··	n=124	n=218	··	n=199	n=172	··	n=105	n=45	··
	Body-mass index, kg/m^2^	26 (5)	26 (6)	0·23	27 (5)	28 (7)	0·10	26 (5)	24 (4)	0·01	24 (4)	23 (3)	0·07
	Fat mass percentage, %	27% (10)	27% (11)	0·56	31% (10)	32% (11)	0·28	26% (9)	22% (8)	<0·0001	23% (9)	21% (8)	0·20

Data are n (%) or mean (SD). Occupation data were missing for one control group participant and three East Village group participants in the social housing group and two participants (control) and one participant (East Village) in the intermediate housing group. Body-mass index data were missing for two participants (East Village) in the social housing group, four participants (control) and two participants (East Village) in the intermediate housing group, and four and two participants in the market rent housing group. Fat mass percentage data were missing for one participant (control) and five participants (East Village) in the social housing group, six and seven participants in the intermediate housing group, and five and two participants in the market rent housing group. Differences between control and East Village groups were tested with χ^2^ or Fisher's exact test for demographic outcomes and *t* tests for physical activity and adiposity outcomes.

We found no effect of moving to East Village on daily steps; daily steps increased by 154 steps (95% CI −231 to 539) after adjusting for sex, age group, ethnic group and housing tenure (table 2). The time spent doing MVPA, either in total or in 10-min bouts or more, daily sedentary time, BMI, and fat mass percentage did not differ between participants who had moved to East Village and those in the control group, including when adjusting for housing group.

**Table 2:** Effect of moving to East Village on physical activity and adiposity outcomes versus control participants after 2 years

		**All housing groups**		**Social housing group**		**Intermediate housing group**		**Market rent housing group**	
		Difference (95% CI)	p value	Difference (95% CI)	p value	Difference (95% CI)	p value	Difference (95% CI)	p value
**Physical activity outcomes (n=762, 290, 335, 137)**									
Daily steps									
	Household (model 1)	192 (−173 to 557)	0·30	–129 (−728 to 469)	0·67	500 (−63 to 1,063)	0·08	160 (−784 to 1,105)	0·74
	Model 1 plus sex, age group, and ethnic group (model 2)	235 (−136 to 605)	0·21	–187 (−803 to 429)	0·55	433 (−175 to 1,042)	0·16	225 (−730 to 1,181)	0·64
	Model 2 plus housing group (model 3)	154 (−231 to 539)	0·43	··	··	··	··	··	··
Daily moderate-to-vigorous physical activity, min									
	Household (model 1)	4 (−16 to 24)	0·73	–12 (−44 to 19)	0·45	21 (−10 to 51)	0·19	8 (−48 to 64)	0·78
	Model 1 plus sex, age group, and ethnic group (model 2)	4 (−16 to 25)	0·67	–19 (−52 to 13)	0·24	12 (−21 to 44)	0·49	13 (−44 to 70)	0·65
	Model 2 plus housing group (model 3)	1 (−20 to 22)	0·91	··	··	··	··	··	··
Daily moderate-to-vigorous physical activity in ≥10-min bouts, min									
	Household (model 1)	3 (−12 to 19)	0·67	–6 (−27 to 14)	0·55	22 (−4 to 47)	0·09	17 (−28 to 63)	0·45
	Model 1 plus sex, age group, and ethnic group (model 2)	4 (−12 to 19)	0·62	–8 (−30 to 13)	0·45	11 (−16 to 38)	0·43	21 (−24 to 67)	0·36
	Model 2 plus housing group (model 3)	6 (−10 to 22)	0·48	··	··	··	··	··	··
Daily sedentary time, min									
	Household (model 1)	–8 (−18 to 2)	0·12	–8 (−28 to 11)	0·39	–2 (−17 to 12)	0·77	3 (−19 to 25)	0·78
	Model 1 plus sex, age group, and ethnic group (model 2)	–8 (−18 to 2)	0·12	–13 (−33 to 7)	0·20	–4 (−19 to 12)	0·64	7 (−15 to 29)	0·54
	Model 2 plus housing group (model 3)	–4 (−15 to 7)	0·45	··	··	··	··	··	··
**Adiposity outcomes (n=822, 327, 358, 137)**									
Body-mass index, kg/m^2^									
	Household (model 1)	0·3 (0·0 to 0·5)	0·06	0·4 (−0·2 to 1·0)	0·16	0·1 (−0·2 to 0·5)	0·54	0·2 (−0·4 to 0·8)	0·52
	Model 1 plus sex, age group, and ethnic group (model 2)	0·2 (−0·1 to 0·5)	0·14	0·2 (−0·4 to 0·8)	0·49	0·1 (−0·3 to 0·5)	0·66	0·2 (−0·4 to 0·8)	0·52
	Model 2 plus housing group (model 3)	0·2 (−0·1 to 0·5)	0·25	··	··	··	··	··	··
Fat mass, %									
	Household (model 1)	0·1% (−0·4 to 0·7)	0·62	–0·1% (−1·1 to 0·8)	0·78	0·1% (−0·6 to 0·8)	0·81	0·3% (−1·0 to 1·7)	0·62
	Model 1 plus sex, age group, and ethnic group (model 2)	0·1% (−0·4 to 0·7)	0·58	–0·3% (−1·3 to 0·7)	0·60	0·2% (−0·6 to 0·9)	0·65	0·4% (−1·0 to 1·8)	0·58
	Model 2 plus housing group (model 3)	0·1% (−0·5 to 0·6)	0·80	··	··	··	··	··	··

Fat mass percentage was missing for one participant in each of the social and intermediate housing groups (n=820 overall for fat mass percentage models).

At a 2-year follow-up, participants who had moved to East Village showed a notable increase in their neighbourhood characteristic scores, indicating a perceived decrease in crime and better quality of their neighbourhood compared with their original neighbourhood at baseline (table 3; appendix pp 5–6). Participants living in East Village lived closer to their nearest park, had better access to public transport, and lived in a more walkable area at follow-up than they did at baseline (appendix pp 5–6). By contrast, we found no changes in the distance to the nearest park, walkability, or access to public transport in participants in the control group who moved elsewhere (appendix pp 7–8). Amongst the control group, those participants who moved to neighbourhoods other than the East Village also perceived improvements in crime safety and neighbour-hood quality compared with control participants who did not move; however, these changes were substantially less than those in the East Village group.

**Table 3:** Within-person change (baseline to follow-up) in neighbourhood perception scores and built environment characteristics

		**All housing groups**		**Social housing group**		**Intermediate housing group**		**Market rent housing group**	
		Mean (95% CI)	p value	Mean (95% CI)	p value	Mean (95% CI)	p value	Mean (95% CI)	p value
**Neighbourhood characteristic scores**									
Control group (n=436, 124, 203, 109)									
	Crime score	0·6 (0·2 to 1·0)	0·004	1·2 (0·4 to 2·1)	0·004	0·1 (−0·5 to 0·7)	0·81	0·9 (0·2 to 1·7)	0·02
	Quality score	0·7 (0·3 to 1·2)	0·0007	1·3 (0·5 to 2·0)	0·001	0·5 (−0·2 to 1·1)	0·14	0·6 (−0·2 to 1·4)	0·15
East Village group (n=441, 220, 174, 47)									
	Crime score	4·6 (4·1 to 5·1)	<0·0001	5·6 (4·9 to 6·3)	<0·0001	3·8 (3·2 to 4·4)	<0·0001	2·7 (1·2 to 4·3)	0·0007
	Quality score	6·8 (6·4 to 7·3)	<0·0001	7·1 (6·4 to 7·8)	<0·0001	6·5 (5·8 to 7·2)	<0·0001	6·8 (5·8 to 7·8)	<0·0001
**Built environment characteristics**									
Control group (n=376, 120, 178, 78)									
	Distance to closest park, m*	6 (−37 to 49)	0·79	–16 (−72 to 39)	0·56	10 (−58 to 78)	0·77	31 (−83 to 144)	0·59
	Access to public transport (PTAL)	–0·2 (−0·3 to 0·0)	0·07	–0·2 (−0·5 to 0·0)	0·07	–0·1 (−0·4 to 0·2)	0·41	–0·2 (−0·6 to 0·2)	0·45
	Walkability	0·3 (0·1 to 0·5)	0·01	–0·2 (−0·5 to 0·2)	0·34	0·6 (0·2 to 1·0)	0·005	0·4 (−0·1 to 0·9)	0·09
	Land use mix	0·02 (0·00 to 0·04)	0·05	–0·03 (−0·05 to 0·00)	0·04	0·04 (0·01 to 0·07)	0·005	0·03 (−0·01 to 0·06)	0·13
	Residential density, 1000 residential units/km^2^	1·9 (1·2 to 2·6)	<0·0001	1·4 (0·7 to 2·1)	0·0001	2·4 (1·2 to 3·5)	<0·0001	1·6 (0·05 to 3·2)	0·04
	Street connectivity	0·0 (−0·1 to 0·1)	0·89	–0·1 (−0·3 to 0·1)	0·17	0·1 (−0·1 to 0·2)	0·42	0·0 (−0·3 to 0·2)	0·89
East Village group (n=414, 216, 160, 38)									
	Distance to closest park, m*	−525 (−565 to −485)	<0·0001	−477 (−527 to −427)	<0·0001	−570 (−633 to −506)	<0·0001	−614 (−812 to −416)	<0·0001
	Access to public transport (PTAL)	1·6 (1·4 to 1·9)	<0·0001	2·5 (2·1 to 2·8)	<0·0001	0·8 (0·4 to 1·3)	0·0002	0·2 (−0·7 to 1·0)	0·66
	Walkability	2·5 (2·2 to 2·7)	<0·0001	2·8 (2·5 to 3·0)	<0·0001	2·2 (1·7 to 2·6)	<0·0001	2·1 (0·6 to 3·7)	0·01
	Land use mix	0·38 (0·36 to 0·40)	<0·0001	0·39 (0·37 to 0·41)	<0·0001	0·38 (0·35 to 0·41)	<0·0001	0·30 (0·20 to 0·39)	<0·0001
	Residential density, 1000 residential units/km^2^	13·2 (12·0 to 14·4)	<0·0001	12·9 (11·4 to 14·4)	<0·0001	12·6 (10·6 to 14·6)	<0·0001	17·4 (12·1 to 22·8)	<0·0001
	Street connectivity	−0·9 (−1·1 to −0·8)	<0·0001	−0·8 (−0·9 to −0·6)	<0·0001	−1·1 (−1·3 to −0·9)	<0·0001	−1·1 (−1·7 to −0·5)	0·0008

Neighbourhood characteristic scores are from exploratory factor analyses on 14 neighbourhood perception items in the study questionnaire. A higher score indicates a perception of less crime in the neighbourhood and a perception that the neighbourhood is of high quality. Neighbourhood perceptions of crime scores range from −10 to 10, and perceptions of quality scores range from −12 to 12. PTAL is a Transport for London score that is used to assess the availability of public transport options; a high score indicates good public transport links. Walkability is the sum of three Z-transformed variables: land use mix, residential density, and street connectivity. Land use mix is the heterogeneity with which five functionally different land uses (residential, commercial, office, entertainment, and institutional) are co-located in space. Values are normalised between 0 and 1, where 0 indicates single use and 1 indicates an even distribution of square footage across the different types of land use. Residential density is the number of residential units per km^2^ of land devoted to residential use, including residential building footprint and attached gardens. Street connectivity is the number of intersections per km of road. PTAL=public transport accessibility level.

* Distance to closest park from a choice of local, district, and metropolitan parks.

Inclusion of an interaction term between the intervention and housing group to allow for potential differential effects was not significant (p>0·1). Restricting analyses to 652 (86%) participants who recorded at least four days of 540-min accelerometry wear or more at baseline and follow-up also showed no significant effect of moving to East Village (versus control participants) on daily steps (difference in steps 324, 95% CI −93 to 741; appendix p 9). We also found no difference when the primary outcome analyses were restricted to weekends (428 steps, −288 to 1144) or weekdays (199 steps, −223 to 620).

Sensitivity analyses to allow the effect of any move to be examined found no change in daily steps between East Village participants and control participants who remained at their baseline address or control participants who moved elsewhere (appendix p 9). There was also no evidence of a difference after removing 21 pregnant women from analyses. Additional adjustment for general health, mental health (depression and anxiety), number of people in the household, marital status, employment status, and length of follow-up showed no effect on the difference between the East Village group and control participants in their change in daily steps (data not shown). The missing data imputation analysis gave similar results to the complete case analysis (appendix p 10).

## Discussion

East Village was assessed to be a more walkable location (with a 2·5-point increase in walkability) than pre-move areas, with greater access to public transport (with a 1·6-point public transport accessibility level increase) and closer proximity to parks (525 m closer). By contrast, no substantive improvements in these measures were observed in control participants who moved elsewhere. East Village was also perceived as having marked improvements in neighbourhood quality. Yet, despite these improvements, at the 2-year follow-up, we found no evidence that participants who moved to East Village increased their daily steps compared with those who did not move to East Village. We also found no difference in time spent in daily MVPA and sedentary behaviour.

A key issue is whether the change in the built environment, in this case East Village, is sufficient and rapid enough to observe changes in health behaviour,^8^ and whether the correct components of the built environment to maximise physical activity were present. Differences in the built environment of East Village were evident by the substantial changes in objective Geographic Information Systems measures of the built environment (including increased walkability, access to public transport, and parks) and notable improvements in neighbourhood perceptions observed among residents, especially those from the social housing group. These findings raise the question of why improvements in physical activity were not observed.

Previous cross-sectional research^7^, ^20^ has shown associations between attributes of the built environment and physical activity, but prospective studies^8^ of housing relocation or neighbourhood change have shown more modest effects. To our knowledge, ENABLE London is the first longitudinal study to use objective measures of physical activity in a cohort that relocated to a new neighbourhood that was specifically designed for healthy active living, rather than relying solely on less reliable self-report.^21^ Daily steps was chosen a priori as the primary outcome, given the focus on examining walkability of the built environment.^12^ We are not aware of any other longitudinal studies with a directly comparable outcome. Despite this difference, some broad comparisons are possible. Although increases in active modes of travel have been observed in previous studies^22^, ^23^, ^24^ (particularly in reported cycling and use of public transport), changes in overall self-reported walking and physical activity have been less apparent.^25^, ^26^, ^27^ However, change in physical activity might be more nuanced. The RESIDential Environment (RESIDE) study,^28^ recruited 1800 people who moved into new homes in Perth (WA, Australia) and, although there were no overall differences in self-reported physical activity, increases in recreational walking and simultaneous decreases in transport-related walking were evident. These differences were attributed to an increase in access to recreational facilities (parks, sports fields, and beaches) and a decrease in access to transport (within a 15-min walk).^29^ Similar compensatory effects in our study might have limited net increases in overall physical activity.

Use of the residential built environment might differ throughout the week. We have previously shown appreciable differences in weekday versus weekend physical activity across housing groups within this cohort at baseline.^17^ However, our prospective findings indicated no change in steps at weekends compared with weekdays. Teasing out the causal relationship between the built environment and health behaviours is complex, and reasons for failing to observe clear effects longitudinally when cross-sectional findings are less equivocal, even within the same study,^27^ remain unclear.^8^ Some systematic reviews^8^ have avoided this issue by excluding longitudinal or experimental studies, raising concerns that such reviews lead to greater uncertainty about biased findings, especially when those living in better neighbourhoods, with built environments that are more conducive to physical activity, are fundamentally different from those who do not. However, it is notable that longitudinal studies can also be prone to selection bias, whereby those who choose to move to better neighbourhoods have different health behaviours compared with those who choose to remain.

The ENABLE London study has several strengths. Our study sought to recruit a cohort who were seeking to move to East Village, in which half moved and half did not, avoiding potential biases in health behaviour when comparing mover with stayer populations. In the absence of randomisation, we believe that our study design offers the next best alternative, providing less biased and stronger evidence about the potential effect of the built environment on health-related behaviours and outcomes. Following the same individuals before and after any move to East Village offers statistical efficiencies, in that individuals act as their own controls, which eliminates confounding factors that do not change within subjects.^30^, ^31^ Moreover, the consistency of effects using 1 or 4 days of objectively measured physical activity allays fears about potential selection effects associated with using fewer days of recording, which were needed to maximise the number of study participants, particularly among the more hard-to-reach social housing group. The use of accurate objective assessment of physical activity is another strength, including assessments of MVPA, which underpin current physical activity recommendations;^3^ this approach allows the potential public health importance of the findings to be gauged. However, we found no effects of moving to East Village on MVPA, suggesting that the change in the built environment had no effect on achievement of physical activity recommendations that focus on greater amounts of activity.^3^ Objective assessment of physical activity reduces measurement error and potential biases arising from self-reported levels of physical activity,^21^ which could be artefactually associated with moving to a new environment that is reported to be aimed at improving healthy active living. The ability to examine different socioeconomic groups allows social inequalities in effects and potential determinants to be examined.^32^ We found no clear differences between housing groups, emphasising that our study was underpowered to formally examine effects on health inequalities.

A key limitation was that our study was only powered to detect an increase of 750 daily steps associated with moving to East Village versus those who did not, so smaller differences could not be formally established.^12^ However, it is important to note that changes of less than 750 steps and more modest changes in MVPA than our study was powered to detect could still constitute clinically significant outcomes, especially when considered at the population level. For example, an increase of 400 steps per day (as observed in our intermediate group) could potentially reduce all-cause mortality by 2% (95% CI 2–4%).^33^ Low sample sizes in our study were due to the modest participation (50–60%), and unforeseen restrictions on recruitment (specifically, on our ability to access the marketing suite when trying to recruit market rent participants).^12^ Moreover, the staged recruitment in which those in the social housing group were moved in first, when East Village was not fully completed, might have resulted in partial exposure to a physical activity promoting environment, and before residential density had reached capacity, which could have reduced the full effect of the exposure.^12^ This possible partial exposure effect was a reason for a 2-year follow-up rather than after 1 year, but this timeframe was also chosen because previous work has suggested that longer durations are needed for habitual health behaviours to develop, and to avoid early so-called honeymoon effects,^34^ or conversely for residents to become fully familiar and make optimal use of their residential area.^35^ The RESIDE study^29^ found larger differences over time, as suburbs developed, new facilities were built, and habits became more established. Further follow-up could plausibly demonstrate beneficial effects but, given the mobility of the cohort and that the study is underpowered, later follow-up is not likely to be possible with a sufficient number of the original cohort participants. Moreover, East Village is developing and the building of high-rise accommodation (in excess of 30 storeys) among the existing 10–12-storey accommodation, and subsequent loss of green space might dampen exposure effects even further by loss of recreational space.^36^ It is plausible that the facilities provided did not meet the needs of the new residents or they did not feel welcome to use those facilities, especially the social housing participants. Qualitative research exploring the lived experience highlighted the benefits of living in East Village, but concerns were raised by those in social housing over the high cost of living, restrictions on the playing times of children, and facilities for young people. These reported issues might help to explain why residents of this new area, particularly those in social housing, did not respond more favourably to an environment designed to be health promoting. Another reason why effects were not more evident might be that London is largely a so-called green city, especially relative to other capital cities,^37^ reducing opportunities to detect change if control participants moved to equally effective spaces. However, the effect was, in fact, marginally strengthened when comparing East Village with control participants who moved elsewhere, and the difference was reduced when comparing East Village with control participants who remained at their baseline address, suggesting that this possibility is not the case. We also found no effect of moving to East Village on adiposity. However, given that physical activity was not increased by moving to East Village, we would not expect changes in adiposity to occur.

Although it remains plausible that the built environment alters physical activity patterns, our study suggests that the effect might be small when people relocate into new, high-density neighbourhoods, with CIs suggesting both increased and decreased daily steps. Hence, the built environment alone is insufficient to induce the change in health behaviour expected by passive means, and more interactive strategies, perhaps in conducive environments such as East Village, are needed. Of note, additional analyses suggest that individual-level changes in the built environment might have greater effects on physical activity than group-level changes, which will be the topic of future work. Potentially, increased accessibility and use of public transport in East Village could favourably affect air quality, leading to more environmentally sustainable communities.^38^ However, these gains need to be weighed against increased individual exposure to air pollution associated with public transport use. There are many discussions globally about the health effects of high-density urban living, particularly high-rise accommodation, and there is an urgent need to mitigate potentially adverse consequences.^39^

Further evidence is required, particularly regarding the factors that resulted in social housing residents who relocated and who decreased their physical activity. Challenges include the need to identify and engage with proposed developments from an early stage, to obtain access to those planning to move long before any move occurs, recruitment of sufficient participants, the unpredictability of creating environmental interventions as planned, and the need for flexible funding to adapt to unforeseen delays, given the researchers' lack of control over any potential build. Only by assimilating evidence from such studies conducted in different types of neighbourhood, which employ common methods that can be directly compared, can the effect of the built environment on physical activity be fully elucidated and understood, to inform planning and provision of housing for optimal health.

## Data sharing

Further details of the ENABLE London study are available from the study website. We welcome proposals for collaborative projects. For general data sharing inquiries, contact Prof Owen.
