# Supplementary material for: The effect of moving to East Village, the former London 2012 Olympic and Paralympic Games Athletes' Village, on physical activity and adiposity (ENABLE London): a cohort study
Source: Lancet Public Health. 2019 Jul 22;4(8):e421–30. doi: 10.1016/S2468-2667(19)30133-1 (PMC6669308; doi:10.1016/S2468-2667(19)30133-1)
Supplement: Supplementary appendix [file mmc1.pdf]

THE LANCET
Public Health

Supplementary appendix

This appendix formed part of the original submission and has been peer reviewed. We post it as supplied by the authors.

Supplement to: Nightingale CM, Limb ES, Ram B, et al. The effect of moving to East Village, the former London 2012 Olympic and Paralympic Games Athletes' Village, on physical activity and adiposity (ENABLE London): a cohort study. *Lancet Public Health* 2019; published online July 22. http://dx.doi.org/10.1016/S2468-2667(19)30133-1.

Appendix

Methods – Derivation of measures of the built environment

Measures of the built environment

Participants were geocoded to the centroid of the footprint of their building of residence at both baseline and follow-up. At baseline and follow-up, each participant was assigned the value of the closest available Public Transport Accessibility Level (PTAL) score (1) from their home address, as a measure of accessibility to public transport. Land-use mix provided a measure of the evenness of distribution of square footage of residential, commercial, office, entertainment and institutional building footprints, street connectivity as the number of 3 or more branch road junctions per street-kilometre, and residential density a measure of the unique residential addresses per squared kilometre of building footprint devoted to residential use. Neighbourhood walkability was derived from a composite score of land-use mix, street connectivity, and residential density within a 1km-street network home address-centred buffer using Ordnance Survey (OS) data.(2) A park proximity variable was also computed at both time points as the shortest street-network distance from the residential addresses to the nearest entrance of the closest park, using data from the Greenspace Information for Greater London database and the London Development Database.(3)

Perceived measures of the built environment

An Exploratory Factor Analysis (EFA) was carried out on the statements assessing perceptions of the local neighbourhood, which were taken from several validated questionnaires.(4) Responses were re-coded from +2 (positive perceptions) to −2 (negative perceptions). Factor loadings were rotated using varimax (orthogonal) rotation. Two scales were produced including a total of 11 statements from a possible 14 (three items did not load strongly onto either of the two factors retained after orthogonal rotation): (i) perceptions of neighbourhood crime (i.e. vandalism, feeling unsafe to walk in neighbourhood, presence of threatening groups) (Cronbach's alpha = 0.87); and (ii) perceptions of neighbourhood quality (i.e. accessible features, attractiveness, enjoyment of living in neighbourhood) (Cronbach's alpha = 0.78). Scores were derived for each scale by summing responses; positive scores indicated lower perceptions of crime and nicer neighbourhoods.(5;6)

References

(1) Transport for London. Accessiblity and connectivity. Available from https://tfl.gov.uk/info-for/urban-planning-and-construction/transport-assessment-guide/transport-assessment-inputs/accessibility-analysis [Accessed November 2018]; 2018.

(2) Ordnance Survey. OS Meridian 2. Availabel from https://digimap.edina.ac.uk/webhelp/os/data_information/os_products/meridian_2.htm [Accessed May 2019]; 2016.

(3) Mayor of London. London Development Database. Available from https://www.london.gov.uk/what-we-do/planning/london-plan/london-development-database [Accessed November 2018]; 2018.

(4) Giles-Corti B, Timperio A, Cutt H, Pikora TJ, Bull FC, Knuiman M, et al. Development of a reliable measure of walking within and outside the local neighborhood: RESIDE's Neighborhood Physical Activity Questionnaire. Prev Med 2006;42:455-9.

(5) Nightingale CM, Rudnicka AR, Ram B, Shankar A, Limb ES, Procter D, et al. Housing, neighbourhood and sociodemographic associations with adult levels of physical activity and adiposity: baseline findings from the ENABLE London study. BMJ Open 2018;8:e021257.

(6) Ram B, Shankar A, Nightingale CM, Giles-Corti B, Ellaway A, Cooper AR, et al. Comparisons of depression, anxiety, well-being, and perceptions of the built environment amongst adults seeking social, intermediate and market-rent accommodation in the former London Olympic Athletes' Village. Health Place 2017;48:31-9.

Supplementary Tables

Table S1. Baseline demographic characteristics, physical activity and adiposity outcomes by housing group for those followed-up and not followed-up.

	**All housing groups (N=1278)**			**Social housing group (N=520)**			**Intermediate housing group (N=524)**			**Market rent housing group (N=234)**		
**N**	Followed-up (N=877)	Not followed-up (N=401)		Followed-up (N=344)	Not followed-up (N=176)		Followed-up (N=377)	Not followed-up (N=147)		Followed-up (N=156)	Not followed-up (N=78)	
	n (%)	n (%)	p-value	n (%)	n (%)	p-value	n (%)	n (%)	p-value	n (%)	n (%)	p-value
**Age, years**												
16-24	179 (20%)	96 (24%)	0.42	65 (19%)	42 (24%)	0.22	68 (18%)	29 (20%)	0.54	46 (29%)	25 (32%)	0.46
25-34	379 (43%)	170 (42%)		93 (27%)	40 (23%)		213 (56%)	89 (61%)		73 (47%)	41 (53%)	
35-49	261 (30%)	106 (26%)		161 (47%)	75 (43%)		83 (22%)	24 (16%)		17 (11%)	7 (9%)	
50+	58 (7%)	29 (7%)		25 (7%)	19 (11%)		13 (3%)	5 (3%)		20 (13%)	5 (6%)	
**Sex**			0.42			0.72			0.68			0.93
Female	495 (56%)	236 (59%)		249 (72%)	130 (74%)		177 (47%)	72 (49%)		69 (44%)	34 (44%)	
Male	382 (44%)	165 (41%)		95 (28%)	46 (26%)		200 (53%)	75 (51%)		87 (56%)	44 (56%)	
**Ethnic group**												
White	437 (50%)	180 (45%)	0.34	63 (18%)	33 (19%)	0.49	261 (69%)	97 (66%)	0.66	113 (72%)	50 (64%)	0.48
Black	212 (24%)	111 (28%)		160 (47%)	91 (52%)		41 (11%)	14 (10%)		11 (7%)	6 (8%)	
Asian	147 (17%)	67 (17%)		78 (23%)	30 (17%)		53 (14%)	24 (16%)		16 (10%)	13 (17%)	
Other	81 (9%)	43 (11%)		43 (13%)	22 (13%)		22 (6%)	12 (8%)		16 (10%)	9 (12%)	
**Occupation, based on National Statistics Social-Economic Coding**												
Higher managerial or professional	425 (49%)	166 (42%)	0.01	47 (14%)	14 (8%)	0.05	270 (72%)	105 (72%)	0.81	108 (69%)	47 (60%)	0.35
Intermediate	123 (14%)	56 (14%)		43 (13%)	19 (11%)		56 (15%)	23 (16%)		24 (15%)	14 (18%)	
Routine or manual	100 (11%)	70 (18%)		72 (21%)	53 (31%)		23 (6%)	11 (8%)		5 (3%)	6 (8%)	
Economically inactive	222 (26%)	105 (26%)		178 (52%)	87 (50%)		25 (7%)	7 (5%)		19 (12%)	11 (14%)	
**Number of children in household**												
None	502 (57%)	234 (58%)	0.31	57 (17%)	33 (19%)	0.049	304 (81%)	132 (90%)	0.04	141 (90%)	69 (88%)	0.78
One	165 (19%)	85 (21%)		106 (31%)	70 (40%)		49 (13%)	10 (7%)		10 (6%)	5 (6%)	
Two or more	210 (24%)	82 (20%)		181 (53%)	73 (41%)		24 (6%)	5 (3%)		5 (3%)	4 (5%)	

Table S1 continued.

N	All housing groups (N=1278) Followed-up (N=877) mean (sd)	Not followed-up (N=401) mean (sd)	p-value	Social housing group (N=520) Followed-up (N=344) mean (sd)	Not followed-up (N=176) mean (sd)	p-value	Intermediate housing group (N=524) Followed-up (N=377) mean (sd)	Not followed-up (N=147) mean (sd)	p-value	Market rent housing group (N=234) Followed-up (N=156) mean (sd)	Not followed-up (N=78) mean (sd)	p-value
**Physical activity (N)**	N=808	N=326		N=311	N=141		N=353	N=116		N=144	N=69	
Daily steps	8919 (3205)	8965 (3488)	0.83	7721 (3243)	8006 (3665)	0.41	9617 (2940)	10024 (3042)	0.20	9792 (2967)	9146 (3314)	0.15
Daily MVPA (mins)	59 (25)	59 (28)	0.95	50 (25)	51 (29)	0.53	64 (23)	67 (25)	0.29	68 (25)	63 (28)	0.13
Daily MVPA in ≥10 minute bouts (mins)	20 (19)	20 (21)	0.90	12 (13)	14 (17)	0.18	24 (19)	25 (19)	0.52	29 (21)	24 (27)	0.14
Daily sedentary time (mins)	585 (83)	562 (90)	<0.001	545 (83)	533 (86)	0.15	607 (72)	578 (91)	<0.001	619 (69)	596 (79)	0.03
**Adiposity (N)**	N=863	N=394		N=342	N=174		N=371	N=144		N=150	N=76	
Body mass index (kg/m^2) [2]	26 (5)	25 (5)	0.25	28 (6)	27 (5)	0.11	25 (5)	24 (4)	0.18	24 (4)	25 (5)	0.19
Fat mass percent [3]	27% (10)	27% (10)	0.76	32% (11)	32% (10)	0.71	24% (9)	23% (9)	0.70	22% (9)	23% (10)	0.68

Footnotes

Data are n (%) or mean (SD). Information on occupation was missing for four participants (followed-up) and three participants (not followed-up) in the social housing group, and three and one participants in the intermediate housing group. Differences between those followed-up and not followed-up were tested with χ^2 or Fisher's exact text for demographic outcomes and *t*-tests for physical activity and adiposity outcomes.

Table S2. Summary data at baseline and follow-up for neighbourhood perception scores and built environment variables, overall and by housing group.

	All housing groups		Social housing		Intermediate		Market-rent	
	Baseline	Follow-up	Baseline	Follow-up	Baseline	Follow-up	Baseline	Follow-up
	Mean (sd)	Mean (sd)	Mean (sd)	Mean (sd)	Mean (sd)	Mean (sd)	Mean (sd)	Mean (sd)
**Neighbourhood characteristic scores [1]**								
**Control group**								
N	436	436	124	124	203	203	109	109
Crime score	2.5 (4.2)	3.2 (4.1)	0.9 (4.7)	2.1 (4.4)	3.2 (3.8)	3.3 (4.0)	3.2 (3.8)	4.2 (3.6)
Quality score	4.5 (4.5)	5.2 (4.1)	3.4 (4.5)	4.7 (4.2)	4.7 (4.5)	5.2 (4.1)	5.1 (4.3)	5.8 (4.0)
**East Village group**								
N	441	441	220	220	174	174	47	47
Crime score	1.6 (4.6)	6.2 (3.3)	0.3 (4.5)	5.9 (3.7)	2.7 (4.1)	6.5 (2.9)	3.7 (4.4)	6.5 (2.8)
Quality score	2.6 (4.4)	9.5 (2.7)	1.7 (4.5)	8.8 (3.1)	3.6 (4.3)	10.1 (2.2)	3.4 (3.8)	10.3 (1.9)
**Built environment characteristics**								
**Control group**								
N	406	385	123	120	188	180	95	85
Distance to closest park (m) [2]	666 (410)	671 (411)	597 (339)	588 (287)	712 (445)	720 (465)	665 (415)	685 (426)
Access to public transport (PTAL) [3]	4.6 (1.8)	4.5 (1.8)	4.5 (1.9)	4.3 (1.8)	4.5 (1.8)	4.4 (1.9)	4.8 (1.8)	4.8 (1.8)
Walkability [4]	0.1 (2.5)	0.4 (2.6)	0.0 (1.9)	-0.2 (2.3)	0.1 (2.7)	0.6 (2.7)	0.4 (2.8)	0.8 (2.3)
Land use mix [5]	0.37 (0.18)	0.39 (0.18)	0.36 (0.15)	0.34 (0.18)	0.36 (0.18)	0.41 (0.19)	0.40 (0.19)	0.43 (0.17)
Residential density [6]	12.0 (5.7)	14.0 (8.1)	10.8 (3.9)	12.3 (5.5)	12.3 (6.1)	14.8 (9.2)	13.2 (6.4)	14.9 (8.2)
Street connectivity [7]	8.7 (1.2)	8.7 (1.1)	8.6 (1.0)	8.5 (1.0)	8.6 (1.3)	8.7 (1.3)	8.8 (1.2)	8.8 (1.0)
**East Village group**								
N	414	441	216	220	160	174	38	47
Distance to closest park (m) [2]	659 (397)	132 (109)	622 (360)	144 (108)	696 (395)	128 (107)	713 (567)	90 (111)
Access to public transport (PTAL) [3]	4.6 (1.9)	6.1 (2.0)	4.1 (1.8)	6.5 (1.9)	5.1 (1.9)	5.9 (2.0)	5.2 (2.0)	5.2 (2.1)
Walkability [4]	-0.1 (2.7)	2.4 (0.8)	-0.6 (2.1)	2.2 (0.8)	0.4 (2.7)	2.5 (0.7)	0.8 (4.6)	3.0 (0.9)
Land use mix [5]	0.37 (0.18)	0.75 (0.08)	0.33 (0.14)	0.72 (0.07)	0.40 (0.19)	0.78 (0.08)	0.51 (0.27)	0.81 (0.08)
Residential density [6]	11.7 (5.9)	25.4 (11.4)	9.8 (4.0)	22.9 (10.7)	13.1 (6.2)	26.0 (10.3)	16.4 (9.0)	34.6 (13.3)
Street connectivity [7]	8.6 (1.2)	7.6 (0.4)	8.5 (1.1)	7.7 (0.5)	8.7 (1.2)	7.7 (0.4)	8.6 (1.9)	7.5 (0.3)

Footnotes

1. Neighbourhood perception scores from exploratory factor analysis on 14 neighbourhood perception items in the questionnaire. A higher score indicates perception of less crime and higher quality in the neighbourhood. Neighbourhood perceptions of crime score ranges from -10 to 10; perceptions of quality score ranges from -12 to 12.
2. Distance to closest park from choice of local, district and metropolitan parks

3. PTAL is a Transport for London (TfL) score assessing the availability of public transport options. A high score indicates good public transport links.
4. Walkability: The sum of three z-transformed variables, land use mix, residential density and street connectivity
5. Land use mix: The heterogeneity with which five functionally different land uses (residential, commercial, office, entertainment and institutional) are co-located in space. Values are normalised between 0 and 1 where 0 indicates single use and 1 indicates a perfectly even distribution of square footage across the different types of land use.
6. Residential density: The number of residential units (RU) per km2 of land devoted to residential use, including residential building footprint and attached gardens, expressed in 1000 RU/km^2
7. Street connectivity: The number of intersections per km of road

Table S3. Within-person change (baseline to follow-up) in neighbourhood perception scores and built environment charateristics for controls who did not move, controls who moved and the East Village group.

	All housing groups			Social housing group			Intermediate housing group			Market-rent housing group		
	mean	(95% CI)	p-value	mean	(95% CI)	p-value	mean	(95% CI)	p-value	mean	(95% CI)	p-value
**Neighbourhood characteristic scores** [1]												
**Controls who stayed at baseline address**		N=205			N=82			N=80			N=43	
Crime score	0.4	(0.0, 0.9)	0.05	0.7	(0.0, 1.4)	0.04	0.3	(-0.4, 1.1)	0.35	0.4	(0.0, 0.9)	0.05
Quality score	0.6	(0.2, 1.0)	0.008	0.9	(0.1, 1.7)	0.03	0.5	(-0.1, 1.1)	0.13	0.6	(0.2, 1.0)	0.01
**Controls who moved from baseline address**		N=231			N=42			N=123			N=66	
Crime score	0.8	(0.1, 1.5)	0.02	2.2	(0.2, 4.3)	0.04	-0.1	(-1.0, 0.8)	0.83	0.8	(0.1, 1.5)	0.02
Quality score	0.9	(0.2, 1.6)	0.02	2.0	(0.3, 3.7)	0.02	0.5	(-0.5, 1.5)	0.33	0.9	(0.2, 1.6)	0.02
**East Village group**		N=441			N=220			N=174			N=47	
Crime score	4.6	(4.1, 5.1)	<0.001	5.6	(4.9, 6.3)	<0.001	3.8	(3.2, 4.4)	<0.001	4.6	(4.1, 5.1)	<0.001
Quality score	6.8	(6.4, 7.3)	<0.001	7.1	(6.4, 7.8)	<0.001	6.5	(5.8, 7.2)	<0.001	6.8	(6.4, 7.3)	<0.001
**Built environment characteristics**												
**Controls who stayed at baseline address**		N=192			N=81			N=74			N=37	
Distance to closest park (m) [2]	-0.3	(-2.1, 1.4)	0.71	-1.6	(-4.5, 1.2)	0.26	-1.1	(-2.6, 0.3)	0.12	4.1	(-2.0, 10.3)	0.18
Access to public transport (PTAL) [3]	No change (zero for all individuals)											
Walkability [4]	0.6	(0.5, 0.6)	<0.001	0.2	(0.1, 0.3)	0.003	0.8	(0.7, 0.9)	<0.001	0.8	(0.7, 1.0)	<0.001
Land use mix [5]	0.03	(0.02, 0.04)	<0.001	-0.01	(-0.02, 0.00)	0.02	0.06	(0.05, 0.07)	<0.001	0.05	(0.04, 0.06)	<0.001
Residential density [6]	2.4	(2.1, 2.8)	<0.001	1.9	(1.5, 2.3)	<0.001	3.2	(2.4, 4.0)	<0.001	2.2	(1.5, 2.9)	<0.001
Street connectivity [7]	0.1	(0.1, 0.1)	<0.001	0.1	(0.0, 0.1)	0.02	0.1	(0.1, 0.2)	<0.001	0.2	(0.1, 0.2)	<0.001

Table S3 continued.

	All housing groups			Social housing group			Intermediate housing group			Market-rent housing group		
	mean	(95% CI)	p-value	mean	(95% CI)	p-value	mean	(95% CI)	p-value	mean	(95% CI)	p-value
**Controls who moved from baseline address**		N=184			N=39			N=104			N=41	
Distance to closest park (m) [2]	12.5	(-76.1, 101.2)	0.78	-46.7	(-222.7, 129.3)	0.59	18.2	(-98.4, 134.8)	0.76	54.6	(-166.1, 275.3)	0.62
Access to public transport (PTAL) [3]	-0.3	(-0.7, 0.0)	0.08	-0.7	(-1.5, 0.1)	0.07	-0.2	(-0.7, 0.3)	0.45	-0.3	(-1.1, 0.5)	0.45
Walkability [4]	0.0	(-0.4, 0.5)	0.89	-0.9	(-1.9, 0.2)	0.10	0.4	(-0.3, 1.0)	0.29	0.1	(-0.8, 1.0)	0.88
Land use mix [5]	0.01	(-0.03, 0.04)	0.70	-0.06	(-0.13, 0.02)	0.14	0.03	(-0.02, 0.08)	0.23	0.01	(-0.06, 0.07)	0.86
Residential density [6]	1.4	(0.1, 2.7)	0.04	0.5	(-1.5, 2.6)	0.61	1.8	(-0.1, 3.7)	0.07	1.1	(-1.8, 4.1)	0.44
Street connectivity [7]	-0.1	(-0.3, 0.1)	0.26	-0.5	(-1.0, 0.0)	0.06	0.0	(-0.3, 0.3)	0.86	-0.2	(-0.6, 0.3)	0.46
**East Village group**		N=414			N=216			N=160			N=38	
Distance to closest park (m) [2]	-525	(-565, -485)	<0.001	-477	(-527, -427)	<0.001	-570	(-633, -506)	<0.001	-614	(-812, -416)	<0.001
Access to public transport (PTAL) [3]	1.6	(1.4, 1.9)	<0.001	2.5	(2.1, 2.8)	<0.001	0.8	(0.4, 1.3)	<0.001	0.2	(-0.7, 1.0)	0.66
Walkability [4]	2.5	(2.2, 2.7)	<0.001	2.8	(2.5, 3.0)	<0.001	2.2	(1.7, 2.6)	<0.001	2.1	(0.6, 3.7)	0.01
Land use mix [5]	0.38	(0.36, 0.40)	<0.001	0.39	(0.37, 0.41)	<0.001	0.38	(0.35, 0.41)	<0.001	0.30	(0.20, 0.39)	<0.001
Residential density [6]	13.2	(12.0, 14.4)	<0.001	12.9	(11.4, 14.4)	<0.001	12.6	(10.6, 14.6)	<0.001	17.4	(12.1, 22.8)	<0.001
Street connectivity [7]	-0.9	(-1.1, -0.8)	<0.001	-0.8	(-0.9, -0.6)	<0.001	-1.1	(-1.3, -0.9)	<0.001	-1.1	(-1.7, -0.5)	<0.001

Footnotes

1. Neighbourhood perception scores from exploratory factor analysis on 14 neighbourhood perception items in the questionnaire. A higher score indicates perception of less crime and higher quality in the neighbourhood. Neighbourhood perceptions of crime score ranges from -10 to 10; perceptions of quality score ranges from -12 to 12.
2. Distance to closest park from choice of local, district and metropolitan parks
3. PTAL is a Transport for London (TfL) score assessing the availability of public transport options. A high score indicates good public transport links.
4. Walkability: The sum of three z-transformed variables, land use mix, residential density and street connectivity
5. Land use mix: The heterogeneity with which five functionally different land uses (residential, commercial, office, entertainment and institutional) are co-located in space. Values are normalised between 0 and 1 where 0 indicates single use and 1 indicates a perfectly even distribution of square footage across the different types of land use.
6. Residential density: The number of residential units (RU) per km2 of land devoted to residential use, including residential building footprint and attached gardens, expressed in 1000 RU/km^2
7. Street connectivity: The number of intersections per km of road

Table S4. Sensitivity analyses for main outcome, daily step counts

Adjustment	All housing groups		Social housing group		Intermediate housing group		Market rent housing group	
	Difference [1] (95% CI)	p-value	Difference [1] (95% CI)	p-value	Difference [1] (95% CI)	p-value	Difference [1] (95% CI)	p-value
**Primary analysis**	**N=762**		**N=290**		**N=335**		**N=137**	
Sex, age group, ethnic group	235 (-136, 605)	0.21	-187 (-803, 429)	0.55	433 (-175, 1,042)	0.16	225 (-730, 1,181)	0.64
Sex, age group, ethnic group, housing group	154 (-231, 539)	0.43						
**Analyses restricted to those with 4 days of recording at baseline and follow-up**	**N=652**		**N=218**		**N=306**		**N=128**	
Sex, age group, ethnic group	357 (-44, 759)	0.08	31 (-661, 724)	0.93	438 (-200, 1,076)	0.18	488 (-527, 1,503)	0.35
Sex, age group, ethnic group, housing group	324 (-93, 741)	0.13						
**Excluding women pregnant at baseline or follow-up**	**N=741**		**N=280**		**N=324**		**N=137**	
Sex, age group, ethnic group	223 (-153, 599)	0.25	-100 (-719, 519)	0.75	409 (-210, 1,028)	0.20	225 (-730, 1,181)	0.64
Sex, age group, ethnic group, housing group	153 (-237, 543)	0.44						
**Analysis limited to weekdays**	**N=755**		**N=286**		**N=333**		**N=136**	
Sex, age group, ethnic group	272 (-133, 677)	0.19	-80 (-778, 619)	0.82	528 (-125, 1,181)	0.11	237 (-812, 1,285)	0.66
Sex, age group, ethnic group, housing group	199 (-223, 620)	0.36						
**Analysis limited to weekend days**	**N=578**		**N=212**		**N=255**		**N=111**	
Sex, age group, ethnic group	410 (-279, 1,099)	0.24	269 (-710, 1,248)	0.59	237 (-929, 1,404)	0.69	337 (-1,732, 2,405)	0.75
Sex, age group, ethnic group, housing group	428 (-288, 1,144)	0.24						
**Control group who stayed at baseline address and Control group who moved from baseline address**	**N=762**		**N=290**		**N=335**		**N=137**	
**East Village vs Controls who stayed at baseline address**								
Sex, age group, ethnic group	94 (-378, 566)	0.70	-254 (-977, 470)	0.49	-85 (-913, 743)	0.84	696 (-637, 2,030)	0.31
Sex, age group, ethnic group, housing group	27 (-454, 507)	0.91						
**East Village vs Controls who moved from baseline address**								
Sex, age group, ethnic group	353 (-91, 797)	0.12	-68 (-978, 843)	0.88	677 (15, 1,339)	0.05	27 (-1,005, 1,060)	0.96
Sex, age group, ethnic group, housing group	265 (-195, 724)	0.26						

Footnotes

1. The change in daily step counts in the East Village group adjusted for the change in the Control group

Table S5. Imputation analyses for main outcome, daily step counts

	All housing groups		Social housing group		Intermediate housing group		Market rent housing group	
**Adjustment**	**Difference (95% CI)**	**p-value**	**Difference (95% CI)**	**p-value**	**Difference (95% CI)**	**p-value**	**Difference (95% CI)**	**p-value**
**Complete case analysis**	**N=762**		**N=290**		**N=335**		**N=137**	
Covariates in model:								
Sex, age group, ethnic group	235 (-136, 605)	0.21	-187 (-803, 429)	0.55	433 (-175, 1,042)	0.16	225 (-730, 1,181)	0.64
Sex, age group, ethnic group, housing group	154 (-231, 539)	0.43						
**Imputations model 1**	**N=808**		**N=311**		**N=353**		**N=144**	
Covariates in model:								
Sex, age group, ethnic group	224 (-150, 597)	0.24	-169 (-789, 451)	0.59	383 (-225, 992)	0.22	219 (-740, 1,178)	0.65
Sex, age group, ethnic group, housing group	140 (-248, 528)	0.48						
**Imputations model 2**	**N=807**		**N=311**		**N=352**		**N=144**	
Covariates in model:								
Sex, age group, ethnic group	239 (-133, 612)	0.21	-166 (-791, 459)	0.60	409 (-194, 1,012)	0.18	244 (-710, 1,199)	0.62
Sex, age group, ethnic group, housing group	155 (-235, 545)	0.44						
**Imputations model 3**	**N=806**		**N=311**		**N=351**		**N=144**	
Covariates in model:								
Sex, age group, ethnic group	241 (-131, 613)	0.20	-166 (-790, 459)	0.60	410 (-190, 1,010)	0.18	241 (-713, 1,196)	0.62
Sex, age group, ethnic group, housing group	157 (-231, 545)	0.43						

Footnotes

1. Imputations were carried out for 46 participants who had accelerometry at baseline but not at follow-up.
2. Model 1 predictors were baseline steps, East Village group, sex, age group, ethnic group and housing group
3. Model 2 predictors were baseline steps, East Village group, sex, age group, ethnic group, housing group and BMI at baseline. One participant had missing BMI at baseline so imputations were only carried out for 45 participants
4. Model 3 predictors were baseline steps, East Village group, sex, age group, ethnic group, housing group and fat mass % at baseline. Two participants had missing fat mass at baseline so imputations were only carried out for 44 participants
